# Vision health perspectives on *Breaking Bad*: Ophthalmic sequelae of methamphetamine use disorder

**DOI:** 10.3389/ftox.2023.1135792

**Published:** 2023-03-08

**Authors:** Ye Huang, Nam V. Nguyen, Danny A. Mammo, Thomas A. Albini, Brent R. Hayek, Brent D. Timperley, Ronald R. Krueger, Steven Yeh

**Affiliations:** ^1^ Department of Ophthalmology and Visual Sciences, Stanley M. Truhlsen Eye Institute, University of Nebraska Medical Center, Omaha, NE, United States; ^2^ Cleveland Clinic, Cole Eye Institute, Cleveland, OH, United States; ^3^ Bascom Palmer Eye Institute, Miller School of Medicine, University of Miami, Miami, FL, United States; ^4^ North Georgia Eye Clinic, Gainesville, GA, United States

**Keywords:** methamphetamine, vision loss, ocular injury, keratitis, neurotoxicity, retinopathy

## Abstract

Methamphetamine use has become a rampant public health issue that not only causes devastating consequences to the user but also poses a burden to surrounding communities. A spectrum of ophthalmic sequelae is associated with methamphetamine use and includes episcleritis, scleritis, corneal ulceration, panophthalmitis, endophthalmitis, retinal vasculitis, and retinopathy. In many instances, prompt recognition of the condition and associated infectious process and early initiation of antimicrobial therapy are crucial steps to preventing vision loss. In this review, we summarize the reported ocular complications that may result from methamphetamine use in addition to several postulated mechanisms regarding the ocular toxicity of methamphetamine. The increasing prevalence of methamphetamine use as a public health threat highlights the need for continued investigation of this ophthalmologic issue.

## 1 Introduction

Methamphetamine, a stimulant with the chemical formula C_10_H_15_N, was initially produced as an amphetamine derivative in the 1890s and was widely used in the 1940–1950s until people became aware of its adverse effects. The U.S. government passed legislation in 1970 labeling amphetamine-type stimulants as controlled substances, limiting medical methamphetamine use, and ensuring close monitoring of the manufacture, prescription, and sale of amphetamine-type stimulants (Vearrier et al., 2012). While this was initially effective in decreasing its use, illegal manufacturing soon emerged in response to the restriction in legal distribution. Since then, steady growth in illicit methamphetamine production and consumption has given rise to a drug use epidemic, a topic which has been depicted in the popular drama television series Breaking Bad (Breaking Bad, 2008). Based on data from the National Survey on Drug Use and Health, the scope of the affected population continues to expand, with approximately 2.5 million people reporting methamphetamine use in 2020 (Administration, 2021). From 2015–2019, there have been upward trends in overdose mortality, risk patterns of methamphetamine use, and increased diversity in populations at risk for methamphetamine use disorder (Han et al., 2021). Following COVID-19 shelter-in-place orders, poison control centers reported an increased call rate for exposure to controlled substances, including opioids and methamphetamine (Maeng et al., 2022). These alarming statistics highlight a growing public health concern and warrant attention from healthcare providers of all disciplines.

Methamphetamine misuse causes various short-term and long-term damages to one’s health. Immediate effects include heightened alertness, euphoria, quickened heartbeat, and increased respiration (NIDA, 2021b). Long-term abuse is deleterious by contributing to development of tolerance with chronic drug use, fueling craving during withdrawal periods. Users may generally require more frequent and higher doses to achieve the original desired effect (Courtney and Ray, 2014). Consequences of long-term methamphetamine use include psychosis, changes in brain structure, deficits in cognitive functioning, memory loss, dental problems (“Meth mouth”), and malnutrition (NIDA, 2022). Increased transmission of hepatitis, HIV, and other infectious diseases is also seen among methamphetamine users (NIDA). Additionally, harmful effects of methamphetamine extend beyond the health of the individual and create a devastating impact on families and communities in the form of increased violence, crime, and corruption (Watt et al., 2014).

This mini-review discusses broader implications of the methamphetamine use epidemic, including the medical, psychosocial, financial, and environmental impacts. We also provide a comprehensive summary of the ophthalmic complications associated with methamphetamine use. The pathophysiology of methamphetamine-related ocular complications has not yet been fully elucidated and is a topic of ongoing investigation.

## 2 Harms of methamphetamine use

### 2.1 Pathophysiology of complications from methamphetamine use disorder

Like other drugs, the mode of methamphetamine consumption can be variable. Smoking is the most common route, followed by injection and inhalation or snorting (Pro et al., 2022). Regardless of how it is consumed, many harmful effects of methamphetamine can be attributed to its sympathomimetic effects, which leads to diffuse vasoconstriction (Hazin et al., 2009). This can present as toxicity of multiple organ systems, some of which are described below.

### 2.2 Medical harm

Methamphetamine elicits a harmful vasoconstrictive effect that can directly cause cardiovascular, cerebrovascular, hepatic, renal, neurologic, and ocular complications. Cardiovascular injuries include arrhythmias or ischemia with or without infarction (Ahmad et al., 2017). Narrowing or frank occlusion of cerebral vessels leads to cerebral ischemia with stroke or hemorrhagic stroke arising from intracerebral hemorrhage (Schep et al., 2010) and may also cause transient cortical blindness (Gospe, 1995; Edinoff et al., 2022). Intoxicated patients who are agitated and hyperthermic are at risk for rhabdomyolysis, which could result in liver damage and renal failure with resultant electrolyte abnormalities (Richards et al., 1999). Individuals may exhibit psychological and neurologic manifestations such as anxiety, depression, psychosis, and deficits in memory and executive functioning (Rusyniak, 2013).

Indirectly, methamphetamine use may contribute to behavioral and immunologic factors that harm the health of an individual. Methamphetamine users engage more frequently in high-risk sex behaviors, increasing avenues for transmission of infectious diseases such as hepatitis and HIV (Hittner, 2016). Behaviors such as unprotected sex, anal sex, use of commercial sex venues, and having multiple concurrent partners are more prevalent in methamphetamine users of the men-who-have-sex-with-men (MSM) population and are associated with a high incidence of sexually transmitted infections (STIs) such as *Chlamydia*, gonorrhea, and syphilis (Taylor et al., 2007; Reback and Fletcher, 2018). Needle or syringe-sharing behavior among users who inject contributes to a higher prevalence of infections (Wada et al., 1999). Methamphetamine may play a role in the immunopathogenesis of HIV and HCV infectivity as well. *In vitro* studies with human cells and animal models have shown that methamphetamine use is associated with higher viral loads, immune dysfunction, antiretroviral resistance, and accelerated progression to AIDS (Marcondes et al., 2010; Harms et al., 2012; Passaro et al., 2015; Skowronska et al., 2018; Liu et al., 2021). Methamphetamine may also damage the integrity of the blood brain barrier, thereby increasing likelihood of CNS involvement during HIV infection (Silverstein et al., 2011). It was also found to enhance replication of HCV in human hepatocytes *in vitro* (Ye et al., 2008).

### 2.3 Social and environmental harm

The methamphetamine epidemic has had significant socioeconomic repercussions, costing the nation an estimated $23.4 billion in 2005 (The RAND Corporation, 2005). This amount was comprised of costs associated with morbidity and mortality, criminal justice and social welfare services, environmental clean-up from methamphetamine production, and lost productivity and quality of life burden due to drug dependence (Nicosia et al., 2009). Methamphetamine users are more likely to have unstable housing, low income, and residence in rural areas (Shearer et al., 2020) with populations of lower socioeconomic status being disproportionately affected. Multiple barriers may prevent these individuals from receiving medical care, as evidenced by higher rates of missed appointments, decreased compliance, and factors that interfere with effective substance use disorder treatment (Marquez et al., 2009; Lai et al., 2020). Additionally, high frequency of co-occurring addiction and mental health problems may lead to a higher risk of treatment non-adherence and missed appointments (Morasco et al., 2014). Children of methamphetamine users are exposed to a myriad of risk factors, including maltreatment, exposure to violence, and criminal behaviors. This environment negatively impacts a child’s psychological development and perpetuates a cycle of neglect and abuse (Gonzales et al., 2010; Messina et al., 2014).

## 3 Relevance to ophthalmology

### 3.1 Overview of ocular signs, symptoms, and complications

Immediately following methamphetamine use, the user may experience blurred vision, mydriasis, disturbances in perception, or visual hallucinations. (Grant and Thomas, 1987; Srisurapanont et al., 2003). Following the acute phase of intoxication, additional ocular complaints may arise if corneal damage occurs. Patients with keratitis may experience days to weeks of decreased vision, eye pain, photophobia, and redness (Franco et al., 2022). They may also describe irritation or a foreign body sensation. Due to hyperstimulation from the drug, the user may fixate on the ocular discomfort and repeatedly rub or pick at the eye, increasing risk for corneal epithelial defects (Poulsen et al., 1996). On exam, focal opacification of the cornea often indicates a corneal ulcer, and severe cases of infection may present with a hypopyon or accumulation of white blood cells that settles in the anterior chamber.

Less frequently reported ocular symptoms arising from the vasoconstrictive effects of methamphetamine include amaurosis fugax, which manifests as short episodes of transient vision loss spontaneously resolving without treatment (Shaw et al., 1985). Similarly, sudden onset of painless vision loss hours after intranasal methamphetamine use may suggest a central retinal artery occlusion (CRAO) or non-arteritic ischemic optic neuropathy (NAION) (Wallace et al., 1992; Wijaya et al., 1999). The presence of blurred vision or visual field defect such as a scotoma may suggest intraretinal hemorrhages (Wallace et al., 1992). Less frequently described ocular findings include painful lid edema, proptosis, chemosis, and corneal melt, which may indicate panophthalmitis (Reed et al., 2021).

### 3.2 Ocular complications associated with methamphetamine use

A spectrum of ophthalmic manifestations associated with methamphetamine use is reported in the literature (Table 1). These infectious or inflammatory processes affect various parts of the ocular anatomy, such as the conjunctiva, sclera, cornea, retina, optic nerve, or orbit.

**TABLE 1:** Spectrum of ophthalmic complications associated with methamphetamine use.

Anatomical structure	Ophthalmic complication
Conjunctiva	Conjunctivitis
Sclera	EpiscleritisScleritis
Cornea	KeratitisKeratolysis or corneal melting
Iris, lens, anterior chamber	MydriasisDecreased accommodation or convergenceHigher risk of angle closure glaucoma
Retina	Amaurosis fugaxRetinal vasculitisRetinal vascular occlusionsIntraretinal hemorrhagesCrystalline retinopathyEndophthalmitis
Optic nerve	Non-arteritic anterior ischemic optic neuropathy (NAION)
Orbit	PanophthalmitisOrbital cellulitis

#### 3.2.1 Conjunctiva and sclera

The vasoconstrictive effect of methamphetamine interferes with ocular perfusion, causing vasculitis, which may manifest as conjunctivitis, episcleritis, or scleritis (Isaak and Liesegang, 1983; Hazin et al., 2009).

#### 3.2.2 Cornea

Postulated mechanisms regarding development of corneal injury among methamphetamine users can largely be grouped into two categories: 1) direct effects and 2) route-related effects. Factors related to methamphetamine production, such as addition of diluting agents or manufacture-related effects, may also contribute to harm caused to the cornea. While each explanation seems plausible, further study is required to determine the exact pathogenesis.

**FIGURE 1:**
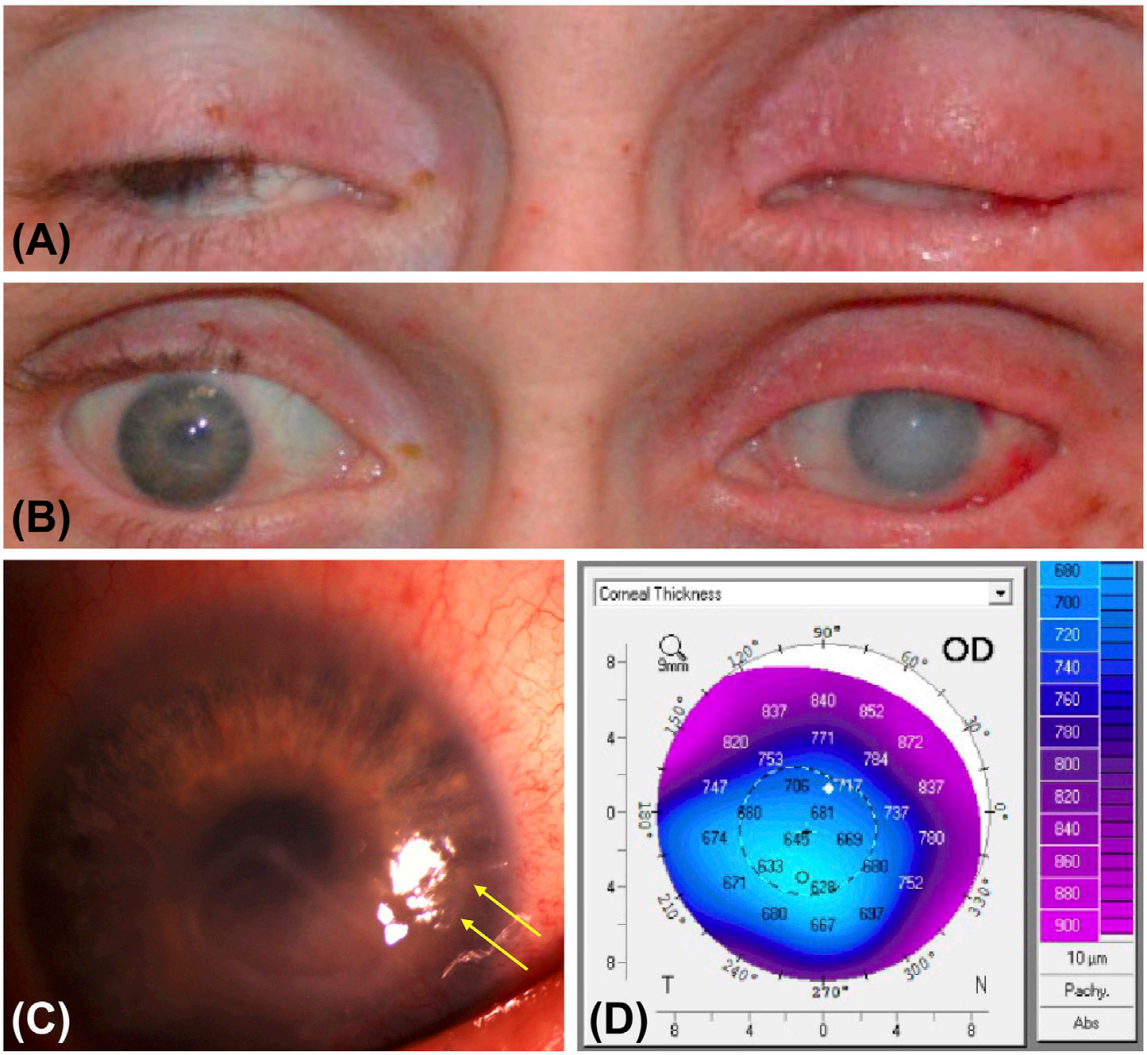
Photographic features and imaging findings of a patient with methamphetamine associated keratitis. **(A)** External photograph of eyes closed show bilateral lagophthalmos, erythema, and lid thickening. There is hypotrichosis of the right eyelid and madarosis of the left eyelid. **(B)** External photograph of eyes open show conjunctival hemorrhage and diffuse corneal edema greater in the left than in the right eye, perilimbal injection in the right eye, and subconjunctival hemorrhage in the left eye. **(C)** Slit lamp photograph of the right eye shows residual corneal ulceration with thickened margins and area of desiccation (yellow arrows). **(D)** Pentacam image of the right eye shows central thinning in the region of ulceration compared to peripheral cornea.

Methamphetamine is a sympathomimetic agent that elevates the pain threshold and disrupts the normal blink mechanism, increasing risk for corneal epithelial insults. With repeated use, damage to dopamine and serotonin receptors could lead to neurotrophic keratitis and microulceration, subsequently presenting as a corneal ulcer (Kroll et al., 2004; Huang et al., 2022) (Figure 1). Methamphetamine is usually sold in the form of hydrochloride salt, which may cause a chemical burn progressing to epithelial defect, a nidus for infection. Toxic damage to the corneal surface may predispose patients to exposure keratopathy and secondary infection (Franco et al., 2022). A case report describes repeated corneal ulcers in a patient recurring concomitantly with periods of heavy methamphetamine smoking (Chuck et al., 1996). Aerosolized and inhaled stimulant use is also associated with keratolysis, a progressive dissolution of the corneal stroma, although the mechanism by which this occurs is unknown (Heer et al., 2020). Interestingly, methamphetamine concentrates in saliva at a tenfold greater concentration than in plasma (Cook et al., 1992). Structural and functional similarities in the lacrimal and salivary glands suggest the possibility for a similar elevation of methamphetamine concentration in the tear film, which could indicate direct corneal toxicity. Risk for drug-to-hand-to-eye exposure is present regardless of the route of methamphetamine consumption. In particular, the fumes from smoking methamphetamine may directly irritate ocular tissues, causing increased eye rubbing and risk of damage to the corneal epithelium.

Addition of diluting agents to methamphetamine samples is meant to increase profits of sale and compounds the likelihood of corneal damage. Anesthetics (lidocaine, procaine) may predispose to ulcer formation, and bases (e.g., bicarbonate, strychnine) can result in alkaline chemical burns. Other sympathomimetics (e.g., caffeine, ephedrine) may augment the vasoconstrictive effect (Poulsen et al., 1996). Hazardous methamphetamine production technique may expose manufacturers and users to harmful contaminants such as metals and solvents, many of which are known corneal toxins, such as mercury (Grant and Thomas, 1987). Manufacturers may also have exposure to corrosives such as sodium hydroxide and sulfuric acid (Burton, 1991; Irvine and Chin, 1991).

#### 3.2.3 Iris, lens, and anterior chamber

Methamphetamine, like other stimulants, commonly causes mydriasis with decreased pupillary reaction (Grant and Thomas, 1987). Decreased accommodation and convergence may also occur, perceived as blurred near vision. Chronic users showed more shallow anterior chamber depth and reduced volume with a higher crystalline lens rise. The combination of these factors with the mydriatic effect of methamphetamine may precipitate risk of angle closure glaucoma (Mahjoob and Heydarian, 2022a). Also, a case of bilateral congenital triangular cataracts in a newborn may have been associated with prenatal methamphetamine exposure from maternal use (Clarke et al., 2009).

#### 3.2.4 Retina and optic nerve

Retinal complications associated with methamphetamine use have also been observed, albeit less frequently than corneal findings. These may include vascular, neural, and infectious implications.

Amaurosis fugax manifesting as episodes of transient vision loss may occur from vasospasm as a direct result of drug use (Shaw et al., 1985). Retinal vasculitis has been observed in association with retinal arteriolar attenuation, optic disc edema, cotton wool spots, and vascular leakage on fluorescein angiogram (Shaw et al., 1985). Retinal emboli are hypothesized to occur from direct intranasal injection *via* retrograde flow of emboli through anastomoses of anterior and posterior ethmoidal arteries and the ophthalmic artery (Byers, 1979; Mabry, 1981). Interestingly, crystalline retinopathy has been reported once as an ocular complication of intranasal methamphetamine use. Postulated causes include drug absorption through vasculature of nasal mucosa or absorption of small particles into pulmonary capillaries (Kumar et al., 2006). Intraretinal hemorrhages and bilateral simultaneous retinal artery and vein occlusions have been reported following intranasal methamphetamine use as well (Wallace et al., 1992; Hazin et al., 2009; Guo et al., 2019). Two potential mechanisms leading to these manifestations may include vasospasm or sudden severe transient hypertension, which can lead to rupture of smaller retinal vessels (Wallace et al., 1992). A case of non-arteritic anterior ischemic optic neuropathy (NAION) was attributed to acute ischemia of short posterior ciliary arteries occurring through vasoconstriction and platelet aggregation contributing to vascular occlusion (Wijaya et al., 1999).

Evidence also suggests methamphetamine has a neurotoxic effect on the retina and may affect retinal morphology. Animal models have demonstrated loss of retinal neurons associated with methamphetamine administration (Yang et al., 2018; Lee et al., 2020). In humans, a statistically significant association was observed between chronic methamphetamine use and retinal nerve fiber layer (RNFL) thickness (Mahjoob et al., 2022) and Bruch’s membrane opening minimum rim width (MRW) (Talebnejad et al., 2020). This phenomenon may result from inflammation and oxidative stress, ultimately leading to visual function disturbances (Yang et al., 2018). Microvascular damage arising from chronic methamphetamine use may also lead to progressive neuronal loss (Guo et al., 2019). Measurement of visual evoked potentials (VEP) is a sensitive tool to assess the functional integrity of the visual pathway, specifically optic nerve activity. Delay in VEP of methamphetamine users has confirmed the detrimental effect of methamphetamine on afferent pathways (Mahjoob and Heydarian, 2022b).

#### 3.2.5 Orbit

Injection drug use increases risk for endophthalmitis, predominantly caused by mycotic and bacterial microorganisms (Poulsen et al., 1996; Kim et al., 2002; Keyashian and Malani, 2007). One case of endophthalmitis secondary to presumed intravenous methamphetamine use required enucleation. Another case of rapidly progressive *Bacillus cereus* panophthalmitis and concomitant orbital cellulitis in an intravenous user required enucleation (Reed et al., 2021).

### 3.3 Ophthalmic implications of methamphetamine production laboratories

In addition to consumption-related smoking or thermal injury, production-related causes of ocular involvement include direct injury, exposure to caustic chemicals during production, or exposure to toxic impurities used to dilute the methamphetamine (Heer et al., 2020). This is especially relevant amidst the rise of illicit methamphetamine production, which uses low-cost ingredients, some of which are dangerous and caustic (Movahedan et al., 2015). A “shake and bake” methamphetamine lab explosion resulted in combined thermal and alkali ocular injury (Chan et al., 2011). While “shake and bake,” also known as the one-pot method for cooking meth, simplifies the cooking process, this particularly dangerous method confers a high risk of fire and explosions with resultant chemical burns, and poisoning (Village, 2022).

Three individuals suffered ocular injuries after using a technique involving combination of ephedrine or pseudoephedrine, sodium or lithium, and anhydrous ammonia (Lee et al., 2003). Ocular complications resulting from an explosion accident include ocular surface failure, symblepharon, ankyloblepharon, and foreshortening of fornices (Movahedan et al., 2015). Nearby individuals such as children and first responders have also been reported to suffer from injury (Watanabe-Galloway et al., 2009; Melnikova et al., 2011). Patients who suffer from methamphetamine production-related burns typically have a larger burn size, higher incidence of inhalation injury, and increased morbidity from injuries (Spann et al., 2006). Patients may be dishonest about the cause of injury, which further confounds the diagnosis. Therefore, it is reasonable to consider the likelihood of a methamphetamine production-related accident when constructing a differential for the presentation of chemical and thermal ocular injuries (Charukamnoetkanok and Wagoner, 2004).

## 4 Future considerations for healthcare providers

The process of diagnosing and effectively caring for methamphetamine-using patients is oftentimes complex. Providers may consider methamphetamine-associated ophthalmic injury as a possibility in patient with uncertain cause, multiple risk factors, and suspicion of patient reluctance to disclose drug use. From an ophthalmologic standpoint, continued methamphetamine use increases predisposition to chronic, recurrent, bilateral corneal ulcers (Chuck et al., 1996). Prompt recognition and initiation of treatment for corneal ulcers and methamphetamine-induced keratitis is crucial to preventing infection progression, which may be unresponsive to aggressive antimicrobial therapy and require more intensive intervention. Continued close monitoring for development or spread of infection is also necessary (Chuck et al., 1996; Poulsen et al., 1996; Hazin et al., 2009).

Providers face a multitude of barriers when treating these patients, including social stigma, limited clinical knowledge, comorbid medical and behavioral health conditions, and a paucity of available treatments (Dunn et al., 2022). Currently, there is no medication approved by the Food and Drug Administration to treat methamphetamine use disorder, although results of a clinical trial utilizing a combination of naltrexone and bupropion are promising (NIDA, 2021a). Behavioral interventions have been efficacious in treating patients with drug use disorders. Implementation of contingency management, which provides incentives to patients dependent on biological confirmation of substance abstinence, has shown improvement in outcomes (AshaRani et al., 2020; Brown and DeFulio, 2020). Other interventions such as cognitive behavioral therapy have also been effective (AshaRani et al., 2020). The Motivational Incentives for Enhancing Drug Abuse Recovery studies performed by the National Institute on Drug Abuse (NIDA) yielded favorable results using incentive-based methods to promote methamphetamine abstinence (Stitzer et al., 2010).

Furthermore, another consideration in caring for these patients is the importance of continuing care, a long-term management approach to maintain abstinence, address relapse, and connect patients to sources of support (McKay, 2021). Continuing care methods used in treatment of substance use disorders include telephone-based continuing care, mindfulness-based relapse prevention, recovery management checkups, and physical health programs. These modalities use strategies such as active patient outreach, incentives, measurement-based care, and adaptive treatment (McKay, 2021). While outcomes are generally beneficial, further analyses should be performed regarding the most effective components of care in improving outcomes of substance addiction.

## 5 Conclusion

The worsening methamphetamine epidemic has brought about reports of the numerous health and societal complications associated with its use. Although ocular complications from methamphetamine use disorder are not as closely studied as other systemic effects, they can rapidly progress to devastating vision loss if not adequately recognized and treated. A range of ocular structures have been implicated, and the reported spectrum of findings includes corneal ulcerations, retinal vascular occlusions, intraretinal hemorrhages, and other retinal findings. While the pathogenesis of these ophthalmic sequelae is not entirely understood, processes involving direct toxicity, vasoconstriction, vasospasm, and transient hypertension are hypothesized to be included. Further investigation of the mechanism of methamphetamine’s ocular toxicity is needed.
